# Exploring healthcare providers’ perceptions regarding the prevention and treatment of chronic pain in breast cancer survivors: A qualitative analysis among different disciplines

**DOI:** 10.1371/journal.pone.0273576

**Published:** 2022-08-25

**Authors:** Yaël Slaghmuylder, Peter Pype, Ann Van Hecke, Emelien Lauwerier

**Affiliations:** 1 Faculty of Medicine and Health Sciences, Department of Public Health and Primary Care, InterProfessional Collaboration in Education, Research and Practice (IPC-ERP), Ghent University, Ghent, Flanders, Belgium; 2 Nursing Department, Ghent University Hospital, Ghent, Flanders, Belgium; 3 Faculty of Psychology and Educational Sciences, Department of Experimental-Clinical and Health Psychology, Ghent University, Ghent, Flanders, Belgium; Unaizah College of Pharmacy, Qassim University, SAUDI ARABIA

## Abstract

**Background:**

The prevention and treatment of chronic pain problems in breast cancer follow-up care require an adequate response from healthcare providers. Generally, this involves the uptake of evidence-based principles regarding pain management in everyday practice. However, despite the extensive literature on effective pain interventions, systematic and coordinated follow-up care is lacking for breast cancer survivors with pain problems in Flanders, Belgium.

**Objective:**

This study aimed to gather insight into healthcare providers’ perceptions of pain prevention and treatment in breast cancer follow-up care, particularly with attention to the multilevel influences on pain follow-up.

**Methods:**

We conducted four online focus groups with twenty-two healthcare providers from different disciplines such as oncologists, pharmacists, nurses, physiotherapists, and psychologists. Data analysis was guided by the Qualitative Analysis Guide of Leuven. This guide is inspired by the constant comparison method, based on Grounded Theory.

**Results:**

The identified influencing factors were thematically grouped into four levels: at the level of the individual healthcare provider, in interaction with the patient, in interaction with colleagues, and at the context level. At each level, we distinguished factors related to healthcare providers’ perceptions such as awareness, knowledge, attitudes, beliefs, experiences, and intentions. For example, because of a lack of knowledge and certain beliefs among healthcare providers, referral to other disciplines often does not happen in the context of pain.

**Conclusion:**

This study points out the need to explore the prevention and treatment of chronic pain after breast cancer from a multidimensional point of view. This involves not only the characteristics of individual healthcare providers but is also inherently interactional and system-like in nature. This analysis provides opportunities for the development of interventions that target the influencing factors of prevention and treatment of chronic pain in breast cancer survivors.

## Introduction

The primary goal of cancer treatment is survival [1]. Due to improved survival rates for breast cancer during recent years, increased attention is given to the quality of life in breast cancer survivors (BCS) [2,3]. This is important as after completion of breast cancer treatment physical and psychosocial disabilities are common [2,4]. One of these disabilities is pain. The prevalence of pain complaints after breast cancer treatment ranges from 13% to 51% [2,5,6]. Ten years after ending primary cancer treatment, 30% of BCSs still experience pain [3]. Pain can have a strong impact on quality of life and is associated with psychosocial aspects such as distress, depression, fear of relapse, fear of pain, loss of sexual desire, problems with body image, helplessness, low self-esteem, difficulties with social relationships, worrying about the end of life, returning to work, and finances [4,6–14]. Pain may become a problem that is inherently complex and multidimensional in nature. Preventing and treating pain therefore require a biopsychosocial approach that involves biological, psychological and social aspects of pain [15].

Biopsychosocial practices are frequently documented in chronic pain literature, especially among persons with chronic non-cancer conditions (e.g. chronic low back pain, rheumatoid arthritis, or fibromyalgia). Pharmacologic interventions often seem to have limited efficacy in resolving chronic pain in these instances [16–19]. The past years, more attention has also been given to a biopsychosocial approach of pain in cancer and cancer survivorship [20,21]. The prevention and treatment of chronic pain in cancer survivors also require a multimodal care plan with pharmacologic, as well as behavioural interventions [22]. Among cancer survivors, BCSs represent one of the largest subgroups [23,24]. There is already a broad basis of evidence for the effectiveness of behavioural interventions in preventing and treating chronic pain in this subgroup, such as cognitive-behavioural therapies [25–29], acceptance and mindfulness-based therapies [30–32], and physical therapies [25,33–39]. These behavioural interventions do not focus on the reduction of pain, but rather aim to stimulate functional recovery [25].

An important element towards effective implementation of a biopsychosocial approach is the behaviour of health care providers (HCP). A biopsychosocial approach to pain requires a coordinated, holistic and interdisciplinary response from different HCPs such as medical specialists, general practitioners (GP), nurses, social workers, physiotherapists, dieticians, and psychologists [10]. There are extensive care programs in place for breast cancer patients following acute treatment and experiencing side effects of their cancer treatment [10]. In contrast to this, it seems that integrated follow-up care is often still lacking for BCSs. For example, after completing breast cancer treatment symptoms such as pain are not always monitored in a standardized manner during follow-up consultations in the hospital, neither is there referral to appropriate care in primary healthcare [40].

In Belgium, a trajectory of a breast cancer patient often starts with a cancer diagnosis in the hospital. According to the diagnosis, a treatment plan is discussed with the patient and afterward implemented in the hospital. After completing their curative cancer treatment, BCSs have to return to the hospital every three to six months for a follow-up consult with one of their medical specialists [41]. Some hospitals also organize a rehabilitation program or offer outpatient consultations with allied health professionals such as psychologists and dieticians. However, these services are often limited in time, and not every hospital has the same resources. Follow-up in the primary healthcare setting can act as an alternative for hospital-based care. Some local initiatives can be observed in practice, but there are no guidelines in place to assure continued care between these two settings [10,24].

So far, the topic of chronic cancer pain is more explored in the context of acute or palliative cancer care [3]. Relatively little research has been conducted about effective ways to organize multimodal approaches to pain in follow-up care [9,42,43]. We would like to gain a better insight into why certain HCPs act accordingly—i.e. use a biopsychosocial approach—in preventing and treating pain in this specific context, and why others do not. Therefore, it is necessary to take into account the attitudes, beliefs, and actions of individual HCPs from different disciplines [44,45]. Existing research about HCPs’ perceptions regarding acute cancer care and follow-up states that HCPs are worried about limited collaboration and communication between different disciplines [46]. HCPs from the same discipline would mainly communicate with each other [47], and they lack certain information about the patient from other HCPs [43]. However, poor communication between HCPs can contribute to fragmented care of cancer survivors [42]. In addition to this, HCPs are not always aware of each other’s role in cancer care [42,46]. Sometimes there is also a lack of trust between different disciplines. For example, some oncologists have limited confidence in a GP’s ability to manage cancer follow-up care [42]. Therefore, HCPs might feel undervalued [48].

We aimed to investigate the following research question by means of a qualitative study. How do HCPs perceive pain prevention and treatment in the context of breast cancer follow-up care? We will be especially attentive to the multilevel influences on pain follow-up as we suppose the problem of inadequate pain prevention and treatment to be inherently complex in nature. Multiple levels such as an individual level, but also social and environmental levels could influence a HCP’s perceptions and adherence to pain prevention and treatment strategies [45,82].

## Materials and methods

### Study sample

We recruited HCPs who work in primary healthcare and/or in a hospital and have experience with follow-up care of BCSs. Cédric Hèle institute—a Flemish knowledge and training centre for psychosocial oncology -, various associations of the included care professions (i.e. Huisartsenvereniging Gent, Vlaamse Beroepsvereniging Zelfstandige Verpleegkundigen, Vereniging Verpleegkundigen Radiotherapie en Oncologie), and the breast clinic and pain clinic of Ghent University Hospital in Flanders, Belgium helped with the recruitment process. These organizations sent out our call for participation by distributing a flyer. Purposeful sampling was applied based on maximum variation according to discipline and setting (hospital versus primary healthcare setting). Thus, we aimed to organize focus groups representing different disciplines and work settings [49–53].

The study was approved by an independent Committee for Medical Ethics affiliated with Ghent University Hospital (reference number BC-09130). The participants signed an informed consent after having received a written explanation of the nature, purpose, and duration of this research.

### Data collection

We conducted this research during the COVID-19 pandemic from February 2021 to April 2021. At the time of data collection, physical contacts had to be limited and HCPs experienced a high workload. Because face-to-face focus groups are time and place-bound and require certain flexibility of the participants, we opted for online focus groups [54,55].

Before the focus groups, participants completed a short questionnaire about gender, age, discipline, years of experience, and place of employment. The multidisciplinary focus groups consisted of two parts. An introductory asynchronous part was organized with the online discussion platform Focus Group It that lasted eleven days, followed by a synchronous part with the video conferencing platform Microsoft Teams [54–61].

We opted for an asynchronous part so that participants would have the time and opportunity to reflect on certain topics regarding breast cancer follow-up care within an anonymous environment and thus reducing the chance of socially desirable responses [55,57]. During the first eight days of the asynchronous focus groups, the moderator asked a new question each day to explore HCPs’ perceptions regarding pain in breast cancer follow-up care. During the last three days, the moderator probed with a few more in-depth questions. The participants got the opportunity to answer each question without specific requirements such as word count or the number of responses. They could also see the answers of other participants and react to each other [55,59]. A semi-structured topic guide was used, based on literature and existing questionnaires (S1 File) [45,62–68]. Additionally, a case about a BCS with pain was offered as a vignette to observe how HCPs would approach follow-up care. The vignette was developed based on real cases in collaboration with clinical nurse specialists in oncology from Ghent University Hospital to ensure the accuracy, reality, relevance, clear language [69], and validity of the case [70].

Afterwards synchronous focus groups took place, during which the major themes from the asynchronous part were discussed in more detail, the answers given were further explored, and group discussion was encouraged [71]. The synchronous focus groups were video recorded and transcribed for analysis. Non-verbal aspects were also noted as an aid in interpreting the data [72].

Because no GPs responded to our call, an extra monodisciplinary focus group with seven GPs was organized. We suspect the low response rate among GPs was due to their important role in the frontline of battling the spread of COVID-19. A combination of an elevated administrative burden, a high workload, and unpredictive hours may have acted as an obstacle to participate. Therefore, we chose to organize a synchronous focus group with Microsoft Teams during a meeting of a Local Quality Group of GPs. A Local Quality Group is a group of colleagues who share and critically assess their medical practice to improve the quality of care [73]. These meetings suffered less from high attrition rates, as GPs need these for better functioning and to meet certain professional requirements.

### Data analysis

Sampling, data collection, and analysis were iterative processes [50,51,74]. Sample characteristics were analysed using the software program IBM SPSS Statistics 26 (SPSS Corporation, Chicago, IL), and described based on descriptive data.

The Qualitative Analysis Guide of Leuven (QUAGOL) was used as a guideline in the analysis of the qualitative data [75]. This guide was inspired by the constant comparison method, based on the Grounded Theory Approach. The proposed method of analysis within this guide was adapted to the current research design [50,51,74], consisting of nine steps. 1) Transcripts of both synchronous and asynchronous focus groups were thoroughly read different times to familiarize with the data and a narrative report was drawn up for each focus group. These reports provide a narrative description of the essence and key storylines of the focus group in answer to the research questions. 2) The first focus group was reviewed independently by three authors of this paper (YS, PP, EL), with relevant data being clustered into concepts. Concrete experiences were replaced by concepts and were presented in a scheme. 3) The obtained conceptual schemes were compared and their suitability verified. Do these concepts reflect the research question, and can these concepts be linked to the data? One common list of concepts was drawn up without imposing a hierarchical order. This non-hierarchical list of concepts was entered into the Nvivo software program [45,75]. 4) The first focus group was again individually reviewed by two authors (YS, EL) and relevant fragments were linked to concepts. If necessary, existing concepts were adapted, new concepts were added, or concepts were split into several sub-concepts. A list of concepts and associated statements was obtained inductively. 5) The lists of the two authors were compared and discussed until a consensus was reached. The third author (PP) was also involved in this comparison to reflect on the coding process. 6) This process was repeated for the other three focus groups, except for conducting a conceptual scheme per researcher. The third author (PP) individually determined the concepts of the other three focus groups. Thereafter, the other authors again linked specific fragments to concepts. 7) One list of concepts was ultimately obtained. These concepts were then individually subdivided into main categories and subcategories. 8) These categories were then discussed within the research team and converted into one common list guided by the Theoretical Domains Framework (TDF) [75]. The TDF comprises cognitive, affective, social, and environmental influences on behaviour. This framework synthesizes 33 theories of behaviour and behaviour change into 14 domains [45,76,77]. TDF is inspirational as an overarching framework, but our study was exploratory and inductive in nature. We first grouped data together and afterward used TDF to define the different clusters. 9) Finally, the fourth author (AVH) read one focus group to check if her findings matched these results.

**Fig 1:**
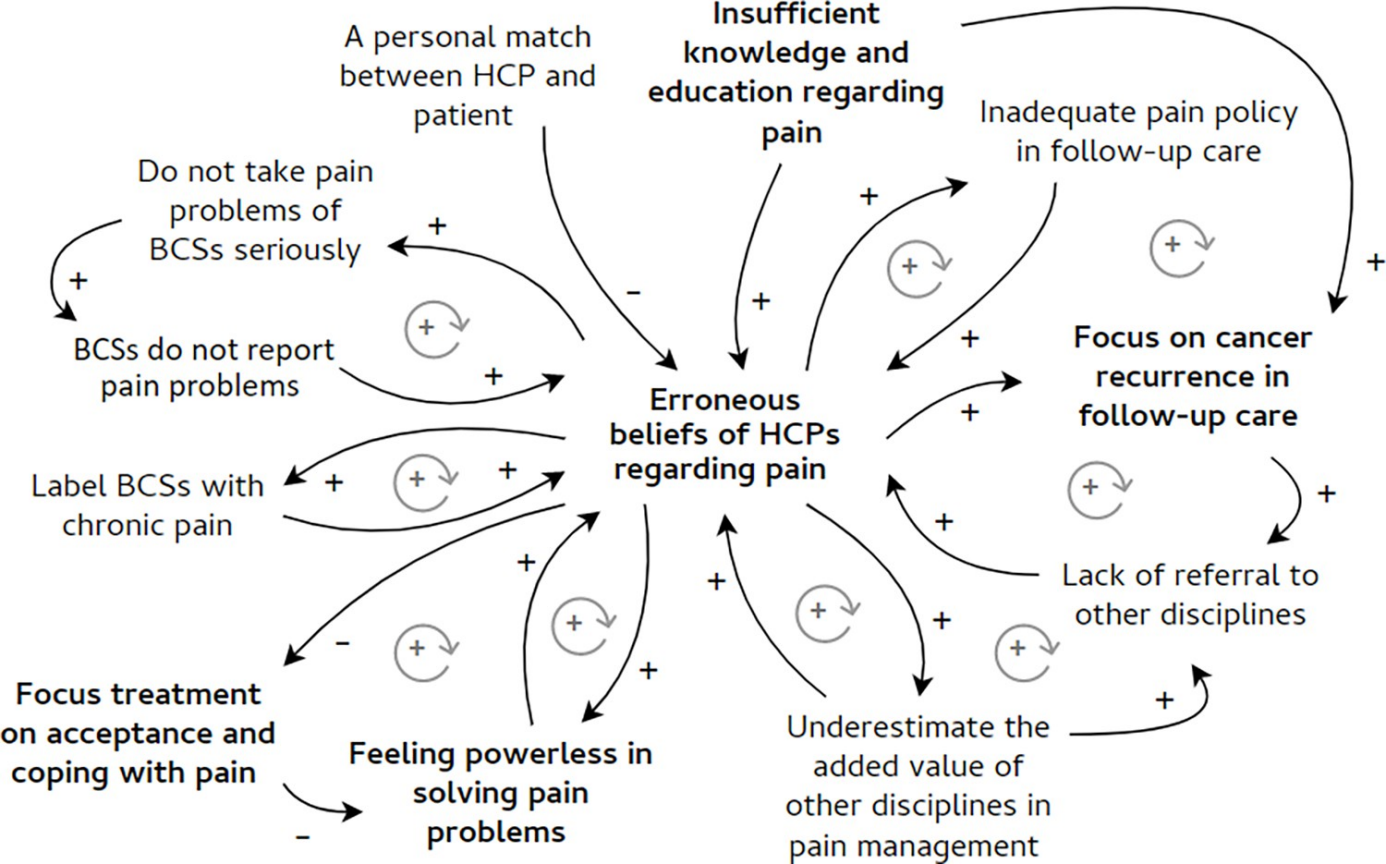
Causal loop diagram. This diagram shows the identified factors at the level of an individual HCP in bold. These factors interact with each other, but also with factors from other levels. Factors related to the interaction between HCPs and patients are presented in the top left corner. Context factors are shown in the top right corner. Factors related to interdisciplinary interactions are displayed in the bottom right corner. The positive arrows represent relationships that reinforce inadequate behaviour of HCPs in preventing and treating pain in breast cancer follow-up care. The negative arrows stand for inhibiting relationships. When a relationship is reciprocal, a circular arrow was added.

To answer the research question, we described the identified factors at four different levels [78]. However, these factors do not always directly influence the behaviour of HCPs in breast cancer follow-up care [45,79]. The participating HCPs also mentioned influences between different factors. Therefore, the relationships between factors at different levels were also explored [80]. To visualize the most prominent relationships, we created a causal loop diagram (Fig 1). Relationships were translated into words-and-arrows diagrams. When a causal link demonstrated a reciprocal relationship, a feedback loop was created. These feedback loops represent relationships and their polarity, shown as circular arrows. A positive polarity (+) stands for a reinforcing relationship, a negative polarity (-) stands for an inhibiting relationship. If all the arrows in the loop are positive or there is an even number of negative arrows, the loop is reinforcing (+). In this study, the polarity shows whether the loop has a facilitating or inhibiting influence on inadequate behaviour of HCPs such as nonadherence to recommended pain prevention and treatment strategies [81]. Finally, significant quotes were added when relevant [75]. Quotes were translated from Dutch to English by one of the authors and proofread by the research team. Quotes from the asynchronous focus groups are referred to as (AS) and from the synchronous focus groups as (S).

### Reliability

Trustworthiness of the data was increased through investigator triangulation. The team approach increased the ability to get to the essence of the data and correct misunderstandings. It provided an in-depth and rich understanding of the research phenomenon. Furthermore, each team member represented a different discipline, which contributed to the quality of the discussions, the integration of different perspectives, and the reliability of the results [75].

## Results

### Study sample

Four focus groups were organized. Each group consisted of four to six HCPs with a total of 22 participating HCPs (S2 File). 17 participants were women and five were men. The age of the participants varied between 25 and 71 years old (average 42 years). The number of years of work experience covered a range of 1.5 to 44 years of experience (average 14 years). The study population represented relevant healthcare professions working in primary healthcare (n = 12), secondary healthcare (n = 6), or tertiary healthcare (n = 2). Two participants worked both in primary healthcare and a general hospital. The represented care professions were an oncologist (n = 1), GPs (n = 7), a pharmacist (n = 1), nurse navigators (n = 2), nurses specialized in breast cancer (n = 2), a nurse specialized in social care (n = 1), physiotherapists (n = 4), psychologists (n = 3), and a psychologist specialized in sexology (n = 1).

The asynchronous focus groups consisted of 183 responses in total. The majority of participants answered every question with an average of eight responses per participant (minimum 6, maximum 16). The video calls of the synchronous focus groups lasted approximately one hour and a half.

### Factors of prevention and treatment of chronic pain in breast cancer follow-up care

We identified various factors that influence the prevention and treatment of chronic pain in breast cancer follow-up care, related to the awareness, knowledge, attitudes, beliefs, experiences, and intentions of HCPs. These factors were distinguished at four levels: at the level of the individual HCP, in interaction with the patient, at the level of the interdisciplinary interaction, and at the context level. First, we described the different levels and their related factors. Second, we explored the complexity of these factors and their influence on pain prevention and treatment. Therefore, we described cross-level relationships between different factors and visualized these relationships in a causal loop diagram (Fig 1).

#### Individual perceptions of HCPs influence pain prevention and treatment

Insufficient knowledge proves to be a common hindering factor in breast cancer follow-up care. Some HCPs feel that they lack knowledge about the causes and management (i.e. prevention and treatment) of chronic pain. They believe that their colleagues have limited knowledge regarding chronic pain as well. Additionally, a few HCPs admit that they do not really know how BCSs are monitored and by whom.

*“Currently*, *I am not well informed about the pain treatments available for breast cancer survivors*. *In my experience*, *these patients are mainly monitored by their general practitioner*.*”*

                     *- Pharmacist (AS)*

Most participating HCPs underestimate the prevalence of pain and pain-related problems in BCSs compared to the numbers reported by previous research. In some types of pain, the pain severity and impact are also underestimated. These types of pain are not described as painful but as complaints.

*“There is certainly a group of breast cancer survivors who experience pain complaints for a long time*. *But*, *I’m rather surprised by the high numbers*. *Pain complaints are rarely reported in my practice*. *I often hear talk about side effects from hormone therapy*. *You might classify this under ‘pain’ because it concerns*, *among other things*, *muscle and joint pain*.*”*

                     *- Psychologist (AS)*

Furthermore, they presume that pain is always more severe after chemotherapy, after a mastectomy, or after an axillary lymph node resection, compared to other treatments. Additionally, they believe that other problems outweigh the pain problems such as fear of relapse.

*“Most women mainly seem to be anxious*, *are afraid of getting sick again*. *They often carry this fear with them every second of the day*. *Also*, *some cannot immediately return to work*, *which reduces their quality of life*. *In terms of pain*, *I have the impression that the pain intensity it is not that bad*.*”*

                     *- Physiotherapist (AS)*

HCPs’ past experiences with preventing and treating pain also play an influencing factor in breast cancer follow-up care. Most HCPs experience pain management as a difficult and complex aspect in follow-up care. They do not always know how to respond to pain problems or do not dare to start a conversation about pain. Some might also create unrealistic expectations about the prognosis of pain (e.g. being pain-free). Eventually, they feel powerless and frustrated when they cannot offer the patient a solution to make them pain-free. A few cope with these feelings by transferring tasks to their colleagues.

*“I recognize that feeling of powerlessness*. *First*, *I listen to the patient but then I quickly suggest that they should talk about their pain problems with their general practitioner or medical specialist*, *or call a nurse specialized in breast cancer*. *There is not much we can do about pain problems*.*”*

                     *- Nurse specialized in social care (S)*

#### Interactions between HCPs and patients influence pain prevention and treatment

In interaction with the patient, a HCP’s awareness of the therapeutic relationship and its impact on pain follow-up is of great importance. For example, HCPs can alter their prejudices towards a patient when they identify themselves with that patient’s situation.

*“Prejudices depend on the therapeutic relationship between patient and physician*. *For example*, *if the patient is a single*, *young mother with two children and the physician herself is divorced*, *then you see—unfortunately—that the physician is more involved*. *In other cases where they feel resistance*, *some complaints remain unheard a little longer or referral is less quick*. *Honestly*, *I think that’s human*. *I don’t think we all have the same connection with every patient*. *… As a psychologist we—I speak for my discipline because I don’t know about other disciplines—I think we are aware if there is something in interaction with the patient*. *What is mine*, *what is the patient’s*? *How can we become aware of these biases to try to rectify them*? *I don’t think everyone is trained to reflect on themselves*. *I don’t think it’s ideal to never again feel irritation in interaction with a patient*, *but rather to become aware of these irritations and to act in a way that care is not compromised*.*”*

                     *- Psychologist (S)*

Another factor is HCPs’ beliefs about the role patients play in their follow-up care. On the one hand, some HCPs state that a patient’s responsibilities in follow-up care must be determined individually. For example, certain patients first need to be motivated before starting a pain treatment. On the other hand, a few HCPs are convinced that the initiative and responsibility in follow-up care always lie with the patient. Patients know the possible pain treatments and are expected to take the first step when needed. They also believe that the success of a pain treatment depends on the motivation of the patient.

*“I may not directly send patients to a psychologist fast enough*. *I think that many patients could benefit from it*, *but usually something is said about psychological guidance in the hospital*. *So they know the option and I am certainly not pushing them*.*”*

                     *- Physiotherapist (S)*

Another belief among HCPs is that pain is linked to a patient’s personality. Pain patients are considered difficult patients who complain a lot or exaggerate the pain problems. Lack of progress or persisting pain problems are attributed to personality traits of a patient rather than situational factors. Several HCPs also perceive that certain patients do not follow their advice or make no effort to understand their explanation about pain.

*“When I told the oncology department head that I was going to a pain clinic*, *the first thing he said was…’You know those pain patients are complainers*, *right*?*’”*.

                     *- Psychologist (S)*

#### Interdisciplinary interactions influence pain prevention and treatment

Participants consider interdisciplinary collaboration important in breast cancer follow-up care. They emphasize the complementary role of their own discipline, but also recognize the added value of other disciplines. However, attitudes towards professional roles and tasks vary among HCPs. On the one hand, certain tasks are supposedly not their own responsibility, but rather that of a colleague. For example, it is the task of GPs to refer to other disciplines.

*“I do recognize that maybe we should refer to physiotherapists a little faster*. *But on the other hand*, *I think that this task belongs to a general practitioner*.*”*

                     *- Psychologist (S)*

On the other hand, they perceive that the added value of other HCPs can be limited in terms of pain prevention and treatment. For example, pain clinics are only used for specific medical interventions, although these interdisciplinary centres aim to support and treat people with chronic pain from a biopsychosocial perspective.

*“The pain clinic consists mainly of anaesthetists who help people with certain back problems*, *as far as I understand*, *more in that context*. *I do not know about pain programs for oncology patients*.*”*

                     *- Psychologist (S)*

Attitudes also differ about who should take the initiative in follow-up care. According to a majority of participants’ experiences, medical specialists like to keep control over pain follow-up. However, they perceive that medical specialists are not always the most suitable persons to take the lead. A few HCPs blame others when things do not work out the way they should but feel they lack leadership to make changes themselves.

*“I experience daily in my position that it is very difficult to be ’allowed’ to help or give advice on pain management for cancer patients*. *Oncologists like to keep this under control*. *We are very rarely asked to come and give advice*.*”*

                     *- Nurse navigator (AS)*

Additionally, a lot of the participating HCPs state that they lack knowledge about the offer and needs of other disciplines. They notice this limited knowledge in their colleagues as well. Furthermore, they do not know the available network of HCPs in their region, which makes interdisciplinary contact and collaboration even more difficult.

*“I am not aware of any psychologists with a specialization in coping with pain. Are there lists of specialized HCPs available anywhere?”*

                     *- General practitioner (S)*

### Context factors influence pain prevention and treatment

We identified different factors of pain prevention and treatment in breast cancer follow-up care at the context level. The participating HCPs perceive that they do not have an impact on these factors. For example, they attribute current shortcomings in follow-up care to a lack of time and solutions for pain. A few HCPs also believe that an follow-up care trajectory includes fewer contact moments, making it impossible to build a trusting relationship with the patient or to actively question pain and psychosocial factors.

*“I believe that I have been trained enough to deal with pain problems, but it just cannot be solved sufficiently. I don’t have a ready-made solution for pain. … You can name those pain problems and give advice. But will that be enough, probably not. I definitely think the bottleneck there is time.”*

                     *- Oncologist (S)*

Another influencing factor is a HCP’s attitude towards which conditions must be met in follow-up care. Some emphasize the need for feasible screening and evaluation instruments, others ask for financial compensation.

*“We always have to be ready*, *committed*, *pay attention to a lot of things*. *All that is possible*, *but we also want a reward for it*. *A serious remuneration*.*”*

                     *- General practitioner (S)*

They also experience that the follow-up care trajectory depends on the availability of resources, such as a rehabilitation program, outpatient consultations in hospitals, and access to medical records. Other influencing factors are the mandates of certain healthcare professions, reimbursement options, legislation, and deontology.

*“It is not in every hospital possible to come for an outpatient consultation with the psychologist after finishing treatment*. *It is nice that we can still see our patients*, *even if they are no longer admitted*. *Another advantage is the continuity of care*. *But in other hospitals this is not always possible*.*”*

                     *- Psychologist (S)*

Additionally, certain characteristics of the healthcare setting play a part such as the physical distance between different services. Furthermore, the internal organization of a setting can influence pain management in follow-up care (e.g. which discipline has the coordinating role in follow-up care, which measures are in place to prevent pain, etc.), Finally, the organizational culture regarding interprofessional communication differs between healthcare settings.

*“Good cooperation and communication are like cross-pollination*. *In the hospital where I currently work*, *I miss communication between different healthcare providers*. *It is not customary here to consult and refer to other healthcare providers*, *in comparison with the hospital I used to work in*.*”*

                     *- Nurse navigator (AS)*

### Relationships between factors

In addition to the above-mentioned factors, cross-level relationships between these various factors were explored and visualized in a causal loop diagram (Fig 1). The relationships are discussed in more detail below.

#### Beliefs of HCPs influence follow-up care at the context level

Erroneous beliefs of individual HCPs regarding chronic pain can implicate that certain types of pain are monitored less structured in follow-up care. A few participating HCPs experience that as a result certain types of pain are not standardly questioned in follow-up care.

*“There has been a very long battle between physicians and nurses*, *discussions about which breast cancer patients should be systematically seen by a psychologist*. *According to the physicians*, *​​people who had a mastectomy suffer more from pain*. *So these patients have to be monitored*. *Meanwhile*, *we—as a psychologist—think that it does not depend on the kind of surgery you get*, *but rather on the person and the situation and many other factors*.*”*

                     *- Psychologist (S)*

#### Beliefs of HCPs influence the interdisciplinary interaction

Erroneous beliefs of HCPs can not only influence the context level, but also the level of interdisciplinary interaction. Because of false beliefs about chronic pain, several HCPs mainly focus on avoiding cancer recurrence during follow-up consultations. When pain is discussed in follow-up care, HCPs suggest imaging tests. They often do not refer in the context of pain, but only when pain occurs in combination with anxiety problems.

*“I often had the feeling that pain was treated medically by the oncologist or radiotherapist*, *who often referred too late to a multidisciplinary pain centre*. *As a result*, *it was possible that patients were constantly looking for a good treatment and did not really know who to turn to*. *As an oncology psychologist*, *I was little involved in such questions*. *Sometimes I was involved when pain coexisted with fear*. *Then I was asked to provide psychological counselling*, *but mainly for the anxiety symptoms*.*”*

                     *- Psychologist (AS)*

Many HCPs experience that when referral does happen, pain medication and invasive surgical procedures are suggested too quickly. The added value of other disciplines in follow-up care is appreciated too little, such as the value of physiotherapists, psychologists and sexologists.

*“As a psychologist/sexologist*, *I often find it difficult to get involved by physicians in the context of the emotional impact of pain problems*. *For example*, *physicians often think of antidepressants without referring patients to first shed some more light on the psychological side*. *I hear a lot that patients just have to accept pain or that pain is treated invasively such as surgery instead of following therapy first*. *This is especially true for sexual pain disorders*. *I experience that gynaecologists quickly suggest applying surgery or laser techniques instead of referring to a sexologist*. *Ignoring the emotional and relational impact ensures that patients cannot gain confidence in their own abilities and body*, *and will more often follow medical treatment*.*”*

                     *- Psychologist/sexologist (AS)*

#### Beliefs of HCPs influence the interaction between HCP and patient

Certain beliefs of HCPs, such as a biomedical perspective on pain, may also influence the interaction with the patient. For example, pain is not always taken seriously when there is no medical evidence available. In addition to this, some HCPs answer pain-related questions poorly or give little attention to the influencing psychosocial factors of pain. Several HCPs experience that BCSs feel that their pain is misunderstood. Therefore, they will no longer report pain problems to HCPs in the future.

*“Patients often indicate that they get the feeling from their medical specialists that the treatment has been successful and that this should be the end of it*. *A few more check-ups and a pharmacological after-treatment and that’s it*. *For many*, *it feels as if the pain complaints they face afterward are only secondary*. *In the end*, *they often no longer indicate to the medical specialists that they are still experiencing pain*. *As a result*, *they will not receive pain treatment and continue their lives in pain*.*”*

                     *- Physiotherapist (AS)*

In addition to negative beliefs about chronic pain, beliefs about the patient also have an influence on the interaction and support of the patient. For example, some patients get labelled as persons who make no effort to follow the advice and treatment of HCPs. As a result, the HCP might question the importance of treating pain in follow-up care, such as providing pain education.

*“It depends a lot on the character of the patient*, *I think*, *how much education helps*. *Education helps people who go along with it and try to understand everything*. *But you also have a lot of patients who don’t make an effort to understand because they don’t want to understand*. *In those cases*, *education really won’t help much*.*”*

                     *- Physiotherapist (S)*

#### The interaction between HCP and patient influences the beliefs of HCPs

Not only the beliefs of HCPs influence the interaction with the patient, but also the other way around. When a HCP’s personality does not align with the patient’s personality, pain is less likely to be believed. In the absence of a personal match between a HCP and a patient, negative beliefs about pain can be reinforced. As a result, BCSs are more likely to be considered difficult patients with a negative influence on the recognition of pain by a HCP.

*“If a physician likes a patient*, *then there are less prejudices*, *also regarding the pain problems*.*”*

                     *- Psychologist (S)*

#### Knowledge and experiences of HCPs influence their beliefs

The participating HCPs link false and biomedical beliefs about pain to the education of physicians, in which little attention is paid to chronic pain after cancer treatment. They believe that physicians have more negative beliefs about pain as a result of insufficient knowledge.

Negative beliefs about pain and pain patients can also be reinforced by experiencing powerlessness.

*“A prejudice can be that someone lingers too long in the illness story or exaggerates the pain out of fear… Sometimes prejudices occur when healthcare providers have the feeling that they do not understand chronic pain properly*, *the feeling of being powerless themselves*.*”*

                     *- Psychologist (AS)*

A participating HCP calls to focus on teaching the patient to cope and accept the consequences of pain, rather than trying to solve the pain. She experiences that this reduces feelings of frustration and powerlessness and gives confidence in her own pain approach.

*“I experience that powerlessness much less*. *I have learned that as psychologists we cannot always directly change the pain*, *but that a lot can be done about limitations*. *That certain ’interventions’ in life (such as dosing*, *activity management*, *sleep hygiene*, *stress management) can nevertheless have an impact on the experience of pain*. *That patients are strongly looking for this*: *’do I have to accept this ’*, *’is this for the rest of my life’*. *That we can play a part in that*: *’look*, *we cannot change the pain directly*, *but that you can bring change with small steps’*. *That is very nice to see*.*”*

                     *- Psychologist (S)*

## Discussion

With this study, we aimed at expanding current knowledge on the influencing factors of the prevention and treatment of chronic pain in breast cancer follow-up by exploring the perceptions of different HCPs. This is of great importance because a systematic and coordinated follow-up is lacking for BCSs with pain problems. We aimed to help inform the future development of organized follow-up care for BCSs with pain problems, by embedding the data collection and analysis within theories of behaviour and behaviour change. We let the TDF inspire us to identify different factors at multiple levels. Determinant frameworks distinguish different domains of influencing factors. These factors act as barriers and facilitators of implementation outcomes such as HCPs’ behaviour. The TDF summarizes factors that could possibly influence a certain behaviour, rather than trying to explain how behaviour is changed [45,82]. These factors are knowledge, skills, social/professional role and identity, beliefs about capabilities, optimism, beliefs about consequences, reinforcement, intentions, goals, memory, attention and decision processes, environmental context and resources, social influences, emotion, and behavioural regulation [45,76,77]. Whereas many theories only focus on individual factors, the TDF acknowledges multiple levels of influence and relationships within and across different levels (e.g. social and environmental levels). However, studies often explore factors individually and assume a linear relationship between the identified factors and implementation outcomes [45,82]. In this study, we tried to take into account that factors may interact with each other, with a possible impact on the relationship between factors and outcomes.

We identified a lack of knowledge and erroneous beliefs regarding chronic pain among HCPs as important factors of pain prevention and treatment in breast cancer follow-up care. These factors also play an influential role according to previous studies about chronic non-cancer pain [15,29,83–88]. For example, the literature states that biomedical beliefs about pain may still prevail among HCPs due to a limited knowledge about chronic pain [84]. A biomedical model of pain assumes that pain is always caused by a physiological pathology [86]. This model does not consider that psychosocial factors can influence the pain experience [88]. HCPs with biomedical beliefs assume that pain is justified when biomedical evidence for the pain is present. When pain is not explained biomedically, negative stereotypes may prevail. One such stereotype is perceiving patients as malingering the pain [15]. Claims are also made as to the level of compliance: a patient did not try hard enough or did not follow the recommendations of the HCP, which is then linked to more adverse outcomes [89].

However, the individual beliefs of HCPs might be influenced by the beliefs of their colleagues. A biopsychosocial approach requires interdisciplinary collaboration in chronic pain prevention and treatment [10]. An interdisciplinary collaboration is an active relationship between HCPs from different disciplines, with a different professional culture and who may represent different organizations or sectors but who work together to provide services for the benefit of patients [44]. Interactions between members of an interdisciplinary team are non-linear. Each member acts autonomously but those individual actions also affect other team members [80]. Research shows that the beliefs of HCPs regarding chronic pain are positively influenced when those HCPs belong to a care network. Being part of a care network is associated with less biomedical beliefs and more biopsychosocial beliefs. It provides more training opportunities and chances to exchange experiences and ideas with other HCPs [90].

Vice versa, perceived social/group norms might also negatively influence the beliefs of a HCP. For example, the language an individual uses in their communication with others is a symbol of their underlying values and beliefs [91]. Language can be a powerful mechanism and negatively influence a colleagues attitudes towards pain and pain patients. According to the allied HCPs in this study, negative beliefs about chronic pain are most prevalent among medical specialists. They experience that medical specialists more often call BCSs with pain problems difficult patients or complainers. When HCPs belief what their colleague says, they might also become convinced that preventing and treating pain after breast cancer treatment is not necessary [92]. In contrast to this, when HCPs do not agree with their colleague, they might lose faith in the collaboration with that colleague. Trust between HCPs is conditional. It can be earned or lost through clinical interactions which demonstrate whether colleagues share similar values [93].

The question remains whether the presence of one (or a few) HCPs with negative beliefs can influence the prevention and treatment of pain in breast cancer follow-up care. The impact of prejudices from a HCP in a dominant position also needs further investigation. Additionally, it is important to gain a better insight into the normative drivers behind interactions between HCPs within an interdisciplinary team. A necessary requirement in interprofessional collaboration is normative integration [94]. Normative integration emphasizes the importance of shared values in co-ordination and collaboration [95]. To offer BCSs integrated follow-up care, different HCPs must share the same values regarding pain prevention and treatment [96]. Furthermore, shared values on an organizational and cultural level are also of great importance for integrated care delivery [95].

How do these limited knowledge and erroneous beliefs of HCPs relate to patient outcomes and quality of care? Although we did not investigate this directly, a few assumptions can be made based on existing pain literature. Research on chronic non-cancer pain states that individuals with chronic pain cannot fulfil the socially acceptable role of a sick person undergoing treatment and returning to normal life. Patients get labelled or attributed a negative stereotype. When these negative stereotypes result in discriminating behaviour towards the patient with pain, there is a possibility of stigmatization [15,97]. Stigmatization involves devaluing and discrediting responses towards individuals with certain characteristics that deviate from the societal norm [84]. It is a social process embedded in social relationships that devalues through conferring labels and stereotyping [97]. When a HCP considers BCSs with chronic pain simulants or exaggerators, they might question the reality or seriousness of the pain problems [15]. As a result, they are less likely to help the patient in pain, possibly resulting in undertreatment of pain [15,84]. Studies on stigma regarding chronic non-cancer pain state that stigmatization by HCPs can influence the pain management but also the behaviour of patients [15]. On the one hand, when patients experience stigmatization, they sometimes internalize the stigma. They start to doubt the credibility of their own pain problems, with a lower physical and emotional quality of life as a result [98]. Additionally, when patients have experienced stigmatization by HCPs in the past, some patients expect to get a similar response from HCPs in the future and will therefore seek less help [99]. On the other hand, when patients feel validated and believed by HCPs, they have a sense of relief, they feel secure, and it gives them courage to move forward [89].

It is important to note that stigmatization manifests itself through mechanisms at an individual, as well as an interpersonal and sociocultural level [87]. For example, individual HCPs might feel that prevailing social/group norms deviate from their own personal beliefs regarding pain. Even when individuals change their negative stereotypes underlying stigma, they might unintentionally not react differently towards the stigmatized group due to these social/group norms [91]. The belief that pain must be solved is also ingrained in our culture. Pain is seen as an alarm signal that occurs when our body is in danger, and needs to be solved. As a result, we are not good at dealing with situations like chronic pain, where there is no ready-made solution available [100].

However, research on stigma in HCPs is scarce. Most often, stigma regarding chronic pain is investigated through patient self-reports and conducted in a non-cancer context [84]. Further research on stigma regarding chronic cancer pain and the effect of stigmatization by HCPs on pain prevention and treatment is necessary.

### Implications

First, the knowledge and beliefs of HCPs about pain and influencing factors should be expanded [86,87,90]. Certain beliefs sometimes derive from a HCP’s education or training in which too little attention is paid to pain mechanisms, adequate medication use, and the importance of an early multidisciplinary approach [83]. Previous studies suggest developing additional education programs for HCPs to gain more knowledge about pain, to shift from a biomedical to a biopsychosocial perspective on pain, and to change the beliefs of HCPs [101–103]. Furthermore, it is of great importance to provide interprofessional education and training for HCPs. Interprofessional education improves collaboration and respect among the members of a care team [104,105]. Professionals also learn more about the different roles and skills of each discipline within a care team [106]. Additionally, other interventions might be necessary to change the attitudes and behaviour of HCPs such as case-based learning, interprofessional shadowing, and clinical experiences [107].

Second, to achieve integrated care for pain problems after breast cancer treatment, the beliefs of different HCPs must be aligned within a care team and across health care settings [96]. Different beliefs can complicate effective collaboration and communication between HCPs [108]. For example, biomedical beliefs are difficult to reconcile with a holistic approach to pain [10]. Furthermore, a biomedical perspective is often not in in accordance with how a BCS perceives their pain problems, and might lead to different expectations regarding pain management between HCPs and BCSs [83]. However, beliefs are often internalized and unspoken. Therefore, it is important to explicitly share beliefs [108]. This calls for interventions that promote reflection and conversation among HCPs [109]. For example, interdisciplinary roundtable meetings could stimulate a shared work culture, give opportunities to address and negotiate diverging beliefs, and achieve a consensus between different goals. During these meetings, all involved stakeholders describe their individual goals and plans for the patient with the aim of developing one joint plan with shared goals [110].

Finally, interprofessional practices in a cancer population are often heterogeneous and dynamic in time. Within a cancer setting, care teams often represent care networks. Not all members of this network work closely together [111]. When developing new interventions, we should take into account that additional conditions might be required such as providing opportunities to share information electronically between different settings [105,111,112].

### Limitations

The recruitment of participants and data collection were organized online. As a result, HCPs with poorer digital literacy might have been less inclined to participate in this study. Due to the COVID-19 pandemic, there was also a high workload for HCPs, which made it hard to find many participants. When necessary, we applied convenience sampling instead of purposeful sampling. For example, only one medical specialist participated in the current study, so the conclusions drawn about biomedical and false beliefs regarding pain among medical specialists cannot be considered completely reliable. In addition to this, the responses of participating GPs might have been different if they were included in a multidisciplinary focus group.

Additionally, this study explored conscious knowledge, attitudes, beliefs, experiences and intentions of HCPs. The question remains what and how other factors play a role in preventing and treating pain of BCSs of which HCPs are unaware of.

## Conclusion

This study points out the need to explore the prevention and treatment of chronic pain after breast cancer in a comprehensive way. This involves not only the characteristics of individual healthcare providers but is also inherently interactional and system-like in nature. Furthermore, this analysis provides opportunities for the development of interventions that target the influencing factors of prevention and treatment of chronic pain in breast cancer follow-up care, aimed at different levels. For example, it is important to address the knowledge of HCPs, but also to stimulate a shift in attitudes and behaviour. Certain skills to collaborate with other HCPs are also necessary. Furthermore, the organization of care must be supportive (e.g. technological tools, and incentives to collaborate between different health care settings).

## Supporting information

S1 File Topic guide for asynchronous focus groups. (PDF)

S2 File Sample characteristics. (PDF)
