# Supplementary material for: Exploring healthcare providers’ perceptions regarding the prevention and treatment of chronic pain in breast cancer survivors: A qualitative analysis among different disciplines
Source: PLoS One. 2022 Aug 25;17(8):e0273576. doi: 10.1371/journal.pone.0273576 (PMC9409579; doi:10.1371/journal.pone.0273576)
Supplement: S1 File — (PDF) [file pone.0273576.s001.pdf]

S1. Topic guide for asynchronous focus groups

Question 1

"A significant proportion of breast cancer survivors report a reduced quality of life and various physical and psychosocial disabilities long after cancer treatment, including pain problems. After a mastectomy, approximately 50% of survivors report chronic pain in the arm, neck, shoulder, armpit, breast, or chest. Between 30 and 66% of survivors treated with hormone therapy experience pain. Even ten years after finishing cancer treatment, 30% of breast cancer survivors still experience above-average pain." Do you recognize this situation in practice? Please explain your answer.

Probes

- What needs do you experience in breast cancer survivors regarding pain?
- How do you identify these needs in breast cancer survivors?
- Do you feel that breast cancer survivors always report their pain problems to you?

Question 2

What do you think is the cause of pain that persists, but for which there is no clear solution?

Probes

- Which aspects can influence the experience of pain in breast cancer survivors?

Question 3

To what extent do you believe that pain management in breast cancer survivors is proceeding as it should? *It is important that you answer this question from the perspective of your own discipline.*

Probes

- What do you believe is the reason for follow-up care not being approached as it should?
- What obstacles do you encounter in the current organization of pain management in breast cancer survivors?
- What aspects do you think could improve the organization of pain management in breast cancer survivors?
- Which characteristics of breast cancer survivors do you think play a role in organizing pain management during follow-up care?
- Do you feel that your discipline, and you personally, can contribute to the organization of pain management in breast cancer follow-up? In which way exactly?
- Which characteristics, in your opinion, at a regional and nation level influence pain management in breast cancer survivors?

Question 4

How do you experience the collaboration between different healthcare providers in pain management of breast cancer survivors?

Probes

- How do you experience the communication between different healthcare providers/different care institutions? How do you communicate with each other?
- How are the tasks and responsibilities of various healthcare providers in follow-up care defined?
- How do you experience the coordination between different healthcare providers/different care institutions?

Question 5

"An is 45 years old. She is married and has a 15 year old son. An works as a manager of a purchasing department. She has a history of breast cancer. In July 2019 she was diagnosed with cancer of her left breast without metastasis. A tumorectomy and sentinel lymph node biopsy were performed. Subsequently, radiotherapy was administered to the left breast. Currently, the cancer treatment is finished. An still follows adjuvant hormone therapy with Zoladex and Femara. During a consultation in the breast clinic, An indicated that she experiences joint pain (diffuse) with a clear decrease of her quality of life. She also experiences pain in her left arm. A bone scan and a CT scan of the chest were performed. These scans showed no clear pathology. An rates her pain on a scale of 0 (no pain) to 10 (worst pain imaginable) as a 7. Because of her pain problems, An is no longer able to perform her daily household tasks. She can no longer work full-time and is trying to return to work progressively. In addition, she is very emotional and also anxious about the impact of pain on her life and her work."
What thoughts do you have when you read An's case? How would you approach An's pain?

<u>Probes</u>

- Why would you choose this approach?
- How would you react to An's pain problems?
- How would you feel if An consulted you with her pain problems?
- What objective(s) would you set for An's pain treatment?
- Why would you choose these objectives?
- What responsibilities do you think An has in her treatment?
- Who else would you involve in An's pain treatment? Think of patients from your practice who are similar to An.
- What obstacles do you think could arise in treating An's pain?

Question 6

How effective would you estimate An's pain treatment?

<u>Probes</u>

- What factors do you think play a role in the success of An's pain treatment?
- What confidence do you have in your own knowledge and skills to treat An's pain?

Question 7

Patients, such as An, sometimes indicate that they do not feel heard by healthcare providers regarding their pain complaints after cancer treatment. They feel that their pain is not taken seriously. Reading this statement, what do you think? Do you recognize this?

Probes

- Have you yourself experienced this reaction from patients before?
- How do you respond to pain complaints reported by breast cancer survivors?
- How do you experience that other healthcare providers respond to pain problems of breast cancer survivors?
- Do you notice prejudices about pain among your colleagues? How?
- How does a care team discuss pain in breast cancer follow-up care? Can you give an example?

Question 8

Finally, we would like to implement a stepped care model that, on the one hand, should ensure that pain is detected more quickly and, on the other hand, that the treatment of (chronic) pain is conducted from a bio-psychosocial perspective. All disciplines necessary to optimally manage pain are involved in this. By stepped care we mean the following: "Stepped care is a way to determine the number and sequence of care interventions needed in order to provide each patient with the necessary level of care. Each patient is offered tailor-made care depending on their needs and risk factors. Thus, every patient does not necessarily receive the entire set of care interventions. It starts with low-intensity interventions and gradually increases to more intense and complex interventions if necessary." Do you have experience with stepped care in breast cancer survivors with pain problems? How do you think stepped care should be implemented?

Probes

- Who should take the lead in this approach?
- What are the conditions for stepped care to run efficiently?
- What communication/division of tasks/coordination is needed to provide good stepped care?
- Which disciplines do you think should be involved in stepped care during breast cancer follow-up?
- What advantages in your opinion does a stepped care model have?
- What obstacles can arise in this approach?
