# Supplementary material for: Exploring healthcare providers’ perceptions regarding the prevention and treatment of chronic pain in breast cancer survivors: A qualitative analysis among different disciplines
Source: PLoS One. 2022 Aug 25;17(8):e0273576. doi: 10.1371/journal.pone.0273576 (PMC9409579; doi:10.1371/journal.pone.0273576)
Supplement: S2 File — (PDF) [file pone.0273576.s002.pdf]

S2. Sample characteristics.

Focus group number	Sex	Age	Discipline	Work setting	Years of work experience
1	Female	31	Physiotherapist	Primary healthcare	7
1	Female	50	Nurse navigator	Secondary healthcare	16
1	Female	34	Oncologist	Tertiary healthcare	5
1	Female	25	Physiotherapist	Primary healthcare	2.5*
1	Female	52	Nurse specialized in breast cancer	Secondary healthcare	2.5*
2	Female	44	Physiotherapist	Primary healthcare	20
2	Female	43	Psychologist	Secondary healthcare	20
2	Female	47	Nurse specialized in social care	Secondary healthcare	22
2	Female	40	Nurse specialized in breast cancer	Secondary healthcare	1.5*
3	Female	28	Sexologist	Both in primary healthcare and a general hospital	4
3	Female	31	Psychologist	Both in primary healthcare and a general hospital	6
3	Female	30	Physiotherapist	Primary healthcare	6.5*
3	Female	27	Pharmacist	Primary healthcare	5
3	Female	33	Psychologist	Tertiary healthcare	10
3	Female	48	Nurse navigator	Secondary healthcare	5
4	Male	59	General practitioner	Primary healthcare	34
4	Female	35	General practitioner	Primary healthcare	7
4	Female	47	General practitioner	Primary healthcare	20
4	Male	-	General practitioner	Primary healthcare	-
4	Male	50	General practitioner	Primary healthcare	25
4	Male	71	General practitioner	Primary healthcare	44
4	Male	64	General practitioner	Primary healthcare	39

**.5 indicates half a year*
